# Supplementary material for: From sequence to enzyme mechanism using multi-label machine learning
Source: BMC Bioinformatics. 2014 May 19;15:150. doi: 10.1186/1471-2105-15-150 (PMC4229970; doi:10.1186/1471-2105-15-150)
Supplement: Additional file 2 — Java code of ml2db. Additional file ml2db_code.tar.gz contains the Java source code to run the multi-label machine learning experiments and save the results to database. The code’s Javadoc is included. [file 1471-2105-15-150-S2.zip › additional file 2/ml2db/utils/doc/uk/ac/standrews/utils/main/database/class-use/DbWriter.html]

Uses of Class uk.ac.standrews.utils.main.database.DbWriter


JavaScript is disabled on your browser.


- Overview
- Package
- Class
- Use
- Tree
- Deprecated
- Index
- Help

- Prev
- Next

- Frames
- No Frames

- All Classes

## Uses of Class uk.ac.standrews.utils.main.database.DbWriter

- Packages that use DbWriter

  | Package | Description |
  | --- | --- |
  |  |  |
  | --- | --- |
  | uk.ac.standrews.utils.main.database |  |
  | uk.ac.standrews.utils.test.database |  |
- - ### Uses of DbWriter in uk.ac.standrews.utils.main.database

    Fields in uk.ac.standrews.utils.main.database declared as DbWriter

    | Modifier and Type | Field and Description |
    | --- | --- |
    |  |  |
    | --- | --- |
    | `DbWriter` | DbManager.`m_dbWriter` the database recorder |

    Methods in uk.ac.standrews.utils.main.database that return DbWriter

    | Modifier and Type | Method and Description |
    | --- | --- |
    |  |  |
    | --- | --- |
    | `DbWriter` | DbManager.`getDbWriter()` |
  - ### Uses of DbWriter in uk.ac.standrews.utils.test.database

    Methods in uk.ac.standrews.utils.test.database that return DbWriter

    | Modifier and Type | Method and Description |
    | --- | --- |
    |  |  |
    | --- | --- |
    | `static DbWriter` | DbWriterTest.`getDbWriter()` |

- Overview
- Package
- Class
- Use
- Tree
- Deprecated
- Index
- Help

- Prev
- Next

- Frames
- No Frames

- All Classes
